# The association of witnessing violence with alcohol and cannabis expectancies among Black, Latinx, and White youth: considering neighborhood context

**DOI:** 10.1007/s00127-025-02939-8

**Published:** 2025-07-02

**Authors:** Carolyn E. Sartor, Nicole Kennelly, Margret Z. Powell, Tammy Chung, Shawn J. Latendresse, Vivia V. McCutcheon

**Affiliations:** 1 Institute for Health, Health Care Policy and Aging Research, Rutgers University, 112 Paterson Street, New Brunswick, NJ 08901, USA; 2 Washington University School of Medicine, St. Louis, MO, USA; 3 Baylor University, Waco, TX, USA

**Keywords:** Substance use expectancies, Witnessing violence, Youth, Black, Latinx

## Abstract

**Purpose:**

To identify associations of past-year witnessing violence with expectancies (anticipated effects) for alcohol and cannabis use in Black, Latinx, and White youth, including possible variations by level of neighborhood advantage and/or race/ethnicity.

**Methods:**

Data were drawn from Follow-up 3 of the Adolescent Brain Cognitive Development Study (n=7,332; weighted distributions: 45.53% girl, 52.33% boy, 2.14% other gender; 11.80% Black, 25.13% Latinx, 63.07% White; weighted mean age=12.94 [SE=0.01]). Positive and negative expectancies were measured using the AEQ-AB for alcohol and the MEEQ-B for cannabis. Neighborhood disadvantage was captured via the Area Deprivation Index (ADI) and analyzed as quartiles. General linear models were fitted to data for each of the four expectancies scores, adjusting for socioeconomic status indicators and testing for witnessing violence by race/ethnicity interactions. Quartile-specific regression coefficients were produced.

**Results:**

Witnessing violence was most prevalent in the highest (most disadvantaged) ADI quartile. Across quartiles, positive alcohol expectancies (βs:0.12–0.26) and positive cannabis expectancies (βs:0.20–0.38) were elevated in youth who witnessed violence; associations were weakest in the lowest quartile. Associations with negative expectancies were non-significant for alcohol and lower only in the second highest quartile for cannabis. All race/ethnicity interactions were non-significant.

**Conclusions:**

Risk conferred by witnessing violence manifests early in the development of alcohol and cannabis use, shaping anticipated positive effects even before many youth initiate use. In addition to lower exposure, residing in an advantaged neighborhood may modestly mitigate risk associated with witnessing violence for developing positive expectancies, underscoring the importance of intervening early with youth in disadvantaged neighborhoods.

## Introduction

### Witnessing violence, alcohol and cannabis use, and neighborhood disadvantage

Despite overall downward trends in recent years, underage drinking and youth cannabis use remain major public health issues, with 52.8% of youth in the U.S. reporting lifetime alcohol use and 36.5% reporting lifetime cannabis use by the end of high school [1]. Witnessing violence has been linked with elevated risk for a range of alcohol and cannabis related behaviors in youth, including early alcohol use [2], frequent binge drinking [3], and cannabis use [3]. Some research further indicates that the association with cannabis use is greater among youth living in disadvantaged neighborhoods [4]– where they are also more likely to be exposed to violence [5, 6]. Neighborhood disadvantage likewise has been linked to increased risk for youth alcohol and cannabis related outcomes in some [7, 8] but not all studies [4, 9, 10, 11]. Further, variations by substance, for example, elevated risk for binge drinking but not cannabis use [12] have been observed. However, among Black youth - the racial/ethnic group most highly represented in this literature - neighborhood disadvantage has been associated with increased risk for both alcohol and cannabis use [7]. Additionally, investigations comparing the magnitude of the associations across racial/ethnic groups have found stronger associations for Black relative to White youth [8, 13]. For instance, reduced odds of binge drinking for Black compared to White youth in highly advantaged neighborhoods has been observed [13], suggesting a protective effect specific to Black youth.

### Alcohol and cannabis expectancies as substance use related outcomes of witnessing violence

Expectancies, commonly categorized as positive or negative, are the anticipated effects of a given substance. Positive expectancies, such as feeling relaxed, are associated with elevated likelihood of using the substance [14, 15, 16], whereas negative expectancies, such as impaired thinking, can deter use [17, 18]. These precursors to use start to develop at a young age [17, 19], before consuming alcohol or cannabis, as young as the preschool years [20]. They are shaped through indirect experiences, such as observing parental alcohol use and consequences [21] and media exposure [22, 23]. Expectancies are robust predictors of early and problem use [14, 15, 24] that are present in both individuals who have used and those who have not yet tried a given substance. They are therefore critical targets for interventions with pre- and early adolescent youth, e.g., providing education around the sedating effects of alcohol to counter the common beliefs about its positive social and arousing effects [25]. Identification of risk factors associated with these predictors of alcohol and cannabis use–specifically, low negative and high positive expectancies - could inform intervention even earlier in the risk pathway. Despite the well-established association of witnessing violence with elevated risk for alcohol and cannabis behaviors [2, 3], expectancies have yet to be considered as a potential substance use related outcome of witnessing violence or exposure to stressful events more broadly.

The notion that expectancies may be shaped by witnessing violence is based on prominent models of stress and substance use. The self-medication hypothesis [26], a widely recognized model of substance use, proposes that individuals use substances to manage negative emotional states, e.g., anxiety following a stressor such as witnessing violence. The decision to use alcohol or cannabis is derived from the belief that it will help to manage that anxiety - a positive expectancy about the effects of alcohol or cannabis use. Tension reduction is in fact a commonly studied specific positive expectancy [27, 28]. According to the stressor-vulnerability model [29], exposure to stressors, such as witnessing violence, elicits coping motives to manage the resulting negative emotional state. Coping motives are highly correlated with positive expectancies [27, 28] and elevated in individuals who have experienced stressful events [31, 32]. Thus, increased positive expectancies may also be elicited by experiencing stressful events that evoke negative emotional states, such as witnessing violence.

### Rationale for the study design and objectives of the study

The aim of the current study was to investigate the association of witnessing violence with alcohol and cannabis expectancies in pre- to early adolescent Black, Latinx, and White youth, including the extent to which these associations vary by level of neighborhood advantage and/or race/ethnicity. We considered potential race/ethnicity and neighborhood differences from the perspective of the minority stress model [33], that youth of color and youth who reside in disadvantaged neighborhoods experience additional systemic stressors such as discrimination. We therefore assessed for distinctions by race/ethnicity within different levels of neighborhood advantage (based on census tract data) to tease apart the potential confounding effects of the overrepresentation of Black and Latinx families in disadvantaged neighborhoods [34, 35]. Likewise, we examined family-level indicators of socioeconomic status (SES; parental education and household income) to address potential confounding by race/ethnicity as a function of the lower average SES of Black and Latinx relative to White families [36]. This approach also allowed us to distinguish between the effects of SES and neighborhood advantage, which are highly correlated but have been shown to impact substance use risk (and thus, potentially expectancies) independently [37, 38].

Based on the evidence for elevated problem alcohol and cannabis use among individuals exposed to violence [2, 3], we hypothesized that witnessing violence would be associated with expectancies reflective of elevated risk - higher positive and lower negative - for both substances. We further hypothesized that the magnitude of associations between witnessing violence and expectancies would be greater among Black and Latinx relative to White youth (across levels of neighborhood advantage) and among youth residing in less versus more advantaged neighborhoods.

## Methods

### Sample and procedures

The Adolescent Brain Cognitive Development (ABCD) Study is an ongoing multi-site longitudinal study of adolescent cognitive development and health in the U.S. [https://abcdstudy.org]. Details of study design and sample ascertainment can be found in prior publications [39]. Briefly, youth aged 9 or 10 and their primary caregiver (hereafter referred to as parent) were recruited from 21 sites between 2016 and 2018. Ascertainment targets were derived using enrollment data from the National Center for Education Statistics and data from the U.S. Census Bureau’s American Community Survey, applying probability sampling to target schools within catchment areas. A centralized Institutional Review Board at the University of California San Diego approved all study protocols. Written informed consent from a parent and assent from the child were obtained at the time of enrollment.

Surveys were administered in person (with the exception of online survey administration during the peak of the COVID-19 pandemic) with the child and parent. Demographic information, such as youth race/ethnicity and SES indicators (i.e., parental education level and household income), were collected in addition to a wide range of health information, including a variety of substance use related factors. Data for the current study were drawn primarily from Follow-up Year 3, release 5, the most recent publicly available data at the time of analysis. Data from baseline and prior follow-ups were used to derive neighborhood and SES variables, as detailed in the Measures section. We used data collected from the racial/ethnic groups with the highest representation in the sample: Black, Latinx, and White, as power to detect race/ethnicity differences would be limited for the underrepresented racial/ethnic groups (e.g., Asian, Native American). Race/ethnicity was categorized by the ABCD Study using parent-reported youth race (Black or African American [hereafter referred to as Black], White) and ethnicity (Hispanic/Latinx, hereafter referred to as Latinx). All youth whose parents identified them as Latinx were categorized as Latinx. Thus, “Black” refers to Non-Latinx Black and “White” refers to Non-Latinx/White. Youth self-reported gender was assessed by asking, “What is your current gender identity?” Response options were “boy,” “girl,” and “another gender, e.g., nonbinary,” (hereafter referred to as “other gender”).

The analytic sample was composed of the 7332 youth with complete data on the core constructs: alcohol expectancies, cannabis expectancies, witnessing violence, and neighborhood disadvantage. A total of 1398 participants were excluded, based primarily on missing geocoded neighborhood data (*n* = 631) and household income (*n* = 619). (Excluded participants had a lower prevalence of witnessing violence and reported lower scores on all expectancies subscales than those in the analytic sample.) Weighted frequencies of race/ethnicity, gender, and SES indicators as well as weighted mean ages are reported by Area Deprivation Index (ADI) quartile (see Measures) in Table 1. The (weighted) gender and race/ethnicity distributions for the full sample were 45.53% girl, 52.33% boy, and 2.14% other gender; 11.80% Black, 25.13% Latinx, and 63.07% White, respectively. The weighted mean age of the full sample was 12.94 (standard error = 0.01).

### Analytic plan

All analyses, including calculations of frequencies and means, were conducted in SAS version 8.3, using SAS SURVEY procedures to account for clustering within family and study site, and applying the ABCD Study sample weights, per ABCD Study analysis guidelines [47]. Consistent with the standard approach in substance use expectancies research, where positive and negative expectancies are conceptualized as related but distinct constructs measured by separate subscales, separate models were estimated for the Positive Expectancies subscale and the Negative Expectancies subscale for both alcohol and cannabis expectancies. To model the association of witnessing violence with expectancies (four separate models for positive and negative cannabis and alcohol expectancies), a general linear model was fitted to the data using the SURVEYREG procedure. Race/ethnicity, age, gender, parental education, and household income were included as covariates. To isolate the independent association of witnessing violence and substance use expectancies, lifetime alcohol use (for alcohol expectancies analyses) and lifetime cannabis use (for cannabis expectancies) were included as covariates to control for the influence of prior use on expectancies. Age was entered as a continuous variable; lifetime alcohol and cannabis use were coded dichotomously. All other covariates were dummy coded, with the following reference categories as the comparison group: White for race/ethnicity, boy for gender, some college for parental education, and $50,000-$99,999 for household income. (All values for categorical variables are shown in Table 1.) Interactions between race/ethnicity and witnessing violence were tested. The DOMAIN subcommand was used to produce regression coefficients specific to each ADI quartile.

### Measures

#### Past-year witnessing violence

Witnessing violence over the past year was assessed using youth reports on the Adverse Life Events Scale (25 items; [40, 41]). Endorsement of any of the items assessing witnessing violent events: “Saw crime or accident,” “Saw or heard someone getting hit,” or “Saw or heard someone being shot at (but not actually wounded) in your school or neighborhood,” resulted in coding as positive for past-year witnessing violence. All others were coded as negative. Weighted frequencies of each event are shown with 95% confidence intervals by ADI quartile in Supplemental Table 1.

#### Alcohol Expectancy Questionnaire-Adolescent, Brief (AEQ-AB)

The AEQ-AB [42] consists of two subscales: Positive Expectancies (4 items, e.g., “Alcohol helps a person relax.” and Negative Expectancies (3 items, e.g., “Alcohol can hurt how well a person gets along with others.”). Items are rated on an ordinal scale, from 1 (Disagree strongly) to 5 (Agree strongly). Each subscale was tested separately for measurement equivalence with respect to race/ethnicity, sex assigned at birth, and intersectional identity (the intersection of race/ethnicity with sex) using moderated non-linear factor analysis. The procedure subsequently produced factor scores for each subscale that were adjusted for non-equivalence. (Details of measurement equivalence methods are reported in Chung et al., 2024 [43]).

#### Marijuana Effect Expectancies Questionnaire-Brief (MEEQ-B)

The MEEQ-B [44] consists of two subscales: Positive Expectancies (3 items, e.g., “Marijuana helps a person relax and feel less tense.”) and Negative Expectancies (3 items, e.g., “Marijuana makes it harder to think and do things.”). Items are rated on an ordinal scale from 1 (Disagree strongly) to 5 (Agree strongly). Moderated non-linear factor analysis was applied to each subscale, resulting in factor scores for each subscale that are adjusted for non-equivalence with respect to race/ethnicity, sex assigned at birth, and intersectional identity.

#### SES indicators and neighborhood disadvantage

Parents self-reported their highest education level and household income. Consistent with prior ABCD publications [45], education level was categorized as a 5-level variable: less than high school, high school, some college, bachelor’s degree, and post-bachelor’s degree and household income was categorized as a 3-level variable: less than $50,000, $50,000–$99,999, and $100,000 or higher. Neighborhood disadvantage was measured via the ADI. The ADI was calculated by linking geocoded data from the parent-reported youth residence at baseline to census-tract data, such as neighborhood level employment, household utilities costs, and housing values [46]. ADI values were calculated by the ABCD Study and included in publicly available data sets, expressed as population-level (national) percentiles (1–100), with higher values indicating greater disadvantage. In this study, we applied the common approach of analyzing ADI percentiles in quartiles: 1: ≤ 25 th, 2: 26 th–50 th, 3: 51 st–75 th, and 4: ≥ 76 th percentiles.

#### Lifetime alcohol and cannabis use

Alcohol use was assessed at each wave by asking participants if they had ever sipped alcohol. Those who responded “Yes” at any wave of data collection were categorized as positive for ever using alcohol; all others were categorized as negative. Cannabis use was assessed at each wave by asking participants if they had ever tried cannabis (even a puff). Those who responded “Yes” at any wave of data collection were categorized as positive for ever using cannabis; all others were categorized as negative. (Items assessing higher levels of use were only administered to participants with positive responses on the trying/sipping items; thus, the selected items captured all ever users.)

## Results

### Past-year witnessing violence, AEQ and MEEQ scores, and lifetime use by ADI quartile

Weighted frequencies of past-year witnessing violence and weighted means of adjusted factor scores for past-year positive alcohol expectancies, negative alcohol expectancies, positive cannabis expectancies, and negative cannabis expectancies are reported in Table 2. Lifetime alcohol use (ever sip) and cannabis use (ever try) are also shown in Table 2. Significant differences across quartiles, reflected in the estimated coefficient for a given quartile being outside of the 95% CIs of the contrasted quartile, are indicated in the far-right column. The prevalence of witnessing violence was significantly lower in Q1 (34.66%) compared to Q4 (39.69%) and in Q2 (33.45%) compared to Q3 (37.48%) and Q4. For positive alcohol expectancies, the mean score for Q1 was significantly higher than for Q2, Q3, and Q4 and the mean score for Q2 was significantly higher than for Q4. Mean significant differences in negative alcohol expectancies scores followed a linear pattern, with Q1 higher than Q2, Q2 higher than Q3, and Q3 higher than Q4. Mean positive cannabis expectancies scores did not differ significantly across quartiles. By contrast, differences in mean negative cannabis expectancies scores were significant, and followed the same linear pattern as alcohol expectancies, with Q1 higher than Q2, Q2 higher than Q3, and Q3 higher than Q4. The lifetime prevalence of alcohol use was significantly higher and the lifetime prevalence of cannabis use was significantly lower in Q1 relative to all other quartiles; the prevalence of lifetime alcohol use was also significantly higher in Q2 than Q4.

### Association of witnessing violence with AEQ and MEEQ scores

Results of the series of regression analyses modeling expectancies scores as a function of witnessing violence, adjusting for race/ethnicity, gender, age, SES indicators, and lifetime cannabis or alcohol use, are summarized in Table 3. Regression coefficients for the witnessing violence variable in the positive alcohol expectancies, negative alcohol expectancies, positive cannabis expectancies, and negative cannabis expectancies models are reported with 95% CIs. Interactions by race/ethnicity were non-significant in all models, so race/ethnicity-specific estimates were not generated. Detailed results, including regression coefficients for each covariate, are reported in Supplemental Tables 2 to 5.

#### Alcohol expectancies

Witnessing violence was associated with elevated positive alcohol expectancies scores in all four ADI quartiles (Q1: 0.12, *p* ≤ 0.001, Q2: 0.17, *p* < 0.0001, Q3: 0.26, *p* < 0.0001, Q4: 0.22, *p* ≤ 0.001). Estimated regression coefficients were significantly lower in Q1 than Q3. There were no significant associations between witnessing violence and negative alcohol expectancies scores in any quartile.

#### Cannabis expectancies

Witnessing violence was associated with elevated positive cannabis expectancies scores in all four ADI quartiles (Q1: 0.20, *p* < 0.0001, Q2: 0.30, *p* < 0.0001, Q3: 0.38, *p* < 0.0001, Q4: 0.21, *p* ≤ 0.001). Estimated regression coefficients were significantly lower in Q1 than Q2 and Q3 and significantly higher in Q3 than Q4. Witnessing violence was associated with lower negative cannabis expectancies scores in Q2 only (−0.08, *p* < 0.05). The association was non-significant in Q1, Q3, and Q4.

## Discussion

The current study examined the association of witnessing violence with alcohol and cannabis expectancies in Black, Latinx, and White early adolescents in the context of neighborhood disadvantage. Building on the evidence that witnessing violence is associated with alcohol and cannabis use [2, 3] and that the magnitude of this association may be elevated in disadvantaged neighborhoods [4], we hypothesized that witnessing violence would be associated with expectancies indicative of high liability to use, that is, higher positive and lower negative expectancies for both substances. We further hypothesized that this association would be higher in disadvantaged neighborhoods as well as among Black and Latinx relative to White youth, as a function of the additional, cumulative stress experienced by marginalized populations [33]. Results revealed partial support for both our primary hypothesis and the hypothesized greater magnitude of associations in less advantaged neighborhoods, distinctions between expectancies for the two substances, and no evidence of race/ethnicity differences within a given level of neighborhood advantage.

### Witnessing violence, alcohol and cannabis use, and expectancies by neighborhood advantage

To provide a context for interpreting the main findings, we consider the results of comparisons of the prevalence of witnessing violence, sipping alcohol, and trying cannabis, in addition to mean expectancies scores by level of neighborhood disadvantage. Consistent with prior studies [5, 6], youth from the most disadvantaged neighborhoods had the highest prevalence of witnessing violence and those from the most advantaged neighborhoods had the lowest. Lifetime cannabis use was very low across ADI quartiles, but it was significantly lower in the most advantaged compared to all three other quartiles, in keeping with some [48] but counter to other [8] findings in the larger literature. Sipping alcohol followed the reverse trend: higher in the most advantaged quartile than the other three, counter to previous reports of higher prevalence of alcohol use in disadvantaged relative to advantaged neighborhoods [8, 49, 50]. This discrepancy is most likely attributable to the (age-appropriate) operationalization of alcohol use in the present study as sipping, which is typically parent sanctioned and in the context of a family event [51, 52]– rather than a deviant behavior– a practice more common in families from socioeconomically advantaged backgrounds [53].

Regarding the observed variation in expectancies by level of neighborhood advantage, we cannot draw comparisons to prior research, as we are unaware of existing studies examining differences in alcohol or cannabis expectancies by neighborhood factors. In their absence, we interpret our findings in the context of the above referenced literature on neighborhood disadvantage and alcohol and cannabis use [8, 48, 49, 50]. Specifically, we address whether patterns in expectancies associated with elevated risk for use– high positive [14, 15, 24] and low negative [17, 18] expectancies– were more commonly observed among youth in disadvantaged relative to advantaged neighborhoods. Negative expectancies for both alcohol and cannabis followed the anticipated pattern: lower in youth from the most disadvantaged relative to other neighborhoods. However, positive alcohol expectancies were highest in the most advantaged group and positive cannabis expectancies did not differ by level of neighborhood advantage. These findings likely reflect the association of use with increased positive expectancies [54, 55, 56]. Cannabis use was very limited and varied little across levels of neighborhood advantage (0.7–2.04%), whereas lifetime prevalence of sipping alcohol ranged from 27.23% for youth in the most disadvantaged neighborhoods to 38.22% for youth in the most advantaged neighborhoods– a substantial enough proportion to detect group distinctions.

### Witnessing violence-expectancies associations in the context of neighborhood disadvantage

We found support across substances and across levels of neighborhood advantage for one component of our primary hypothesis, that positive expectancies would be higher among youth who had witnessed violence. As links between stressors such as witnessing violence and expectancies have not previously been studied, we formulated this hypothesis based on the literature linking witnessing violence to elevated risk for alcohol and cannabis use [2, 3]. That is, we proposed that associations of witnessing violence with use would extend to precursors to use that robustly predict early and problem use of cannabis and alcohol [14, 15, 17, 18, 23], i.e., positive expectancies. The counterpart to high positive expectancies in a high liability profile is low negative expectancies. Thus, the second component of the primary hypothesis was that witnessing violence would be associated with lower negative expectancies, for which we found no support for alcohol and very limited support for cannabis (second ADI quartile only).

The specificity of the witnessing violence-expectancies link to positive expectancies may be understood at a mechanistic level under the stressor-vulnerability model [29]. Positive expectancies include anticipated effects directly related to experiencing stress, such as tension reduction, which have been found to elicit coping motives following a stressor [30]. Witnessing violence may evoke a drive to cope with the resulting stress and thus increase salience of potential coping mechanisms, such as using cannabis or drinking alcohol. By contrast, there is no clear direct link between experiencing a stressor and holding a less negative perception of using alcohol or cannabis.

The second hypothesis, which addressed variation in magnitude of the association between witnessing violence and expectancies also had two components; we hypothesized stronger associations among Black and Latinx youth and stronger associations among youth from disadvantaged neighborhoods. With respect to potential race/ethnicity differences, our modeling approach, which produced ADI quartile-specific results, revealed no within-quartile distinctions by race/ethnicity in the strength of associations for any alcohol or cannabis expectancies. These findings indicate that once neighborhood context and family-level SES were taken into account, there were no additional factors for which race/ethnicity may have served as a marker (i.e., social determinants of health) impacting the strength of the association between witnessing violence and alcohol or cannabis expectancies.

Regarding neighborhood disadvantage, given the null findings across quartiles for negative expectancies, we limit our interpretations to positive alcohol expectancies and positive cannabis expectancies. In the only known prior study to report on variation by neighborhood disadvantage in the relationship between youth violence exposure and any substance use related constructs, Fagan and colleagues [4] found that the elevated risk for cannabis use– but not alcohol use– was greater for adolescents in disadvantaged neighborhoods. By contrast, results from the current study did not differ meaningfully between the two substances. For both alcohol and cannabis expectancies, our findings broadly supported weaker associations with witnessing violence among youth in the most advantaged neighborhoods. However, the trend was not linear; that is, we did not consistently observe increasing magnitude of associations with increasing neighborhood disadvantage. In fact, associations between witnessing violence and positive expectancies were no greater for youth living in the most disadvantaged neighborhoods than youth in any more advantaged neighborhoods for either alcohol or cannabis. Thus, our findings may be best interpreted as advantaged neighborhoods mitigating risk rather than disadvantaged neighborhoods exacerbating risk conferred by witnessing violence for anticipating positive effects of alcohol or cannabis.

### Limitations

Certain limitations should be kept in mind when interpreting study findings. First, as our sample was composed of middle-school-aged youth broadly categorized as Black, Latinx, and White, inferences cannot be made about other racial/ethnic groups, variation within each category of race/ethnicity, or other age groups. Expectancies change with age [57, 58] as well as experience with substances [54, 55, 56], and as previously noted, alcohol use was largely parent-sanctioned sipping and cannabis use was very limited in this sample. Additionally, as previously noted, the subset of participants excluded due to missing data on core constructs had a lower prevalence of witnessing violence and reported lower scores on all expectancies subscales than those in the analytic sample. It is possible that including this subset characterized by lower endorsement of witnessing violence and lower expectancies scores could have resulted in a weaker estimated association between witnessing violence and expectancies in the full sample. However, the extent to which these associations between witnessing violence and expectancies may differ between the included and excluded subsets of participants cannot be determined. Further, our operationalization of witnessing violence is not comprehensive and the events captured could vary in frequency or severity. Similarly, endorsement of seeing someone getting hit and seeing or hearing someone getting shot were less common in the most advantaged and more common in the most disadvantaged neighborhoods (see Supplemental Table 1); thus, the severity of events captured under the term “witnessing violence” varied slightly across level of neighborhood advantage.

### Conclusions and implications

Our findings indicate that during the pre- to early adolescent years, witnessing violence is a relatively common experience, particularly for youth living in disadvantaged neighborhoods, who also hold lower negative expectancies about the effects of alcohol and cannabis relative to youth in more advantaged communities. Our findings further suggest that despite being a commonplace event, witnessing violence is associated with increased anticipated positive effects of alcohol and cannabis, which is concerning, as positive expectancies can lead to using alcohol or cannabis to cope with negative affect [27, 28]. In the present study, youth living in more advantaged neighborhoods were not only less likely to report witnessing violence but the impact on positive expectancies appeared to be lower than for youth living in more disadvantaged neighborhoods. These findings highlight the importance of attending to more vulnerable populations at a young age, given the evidence that risk conferred by witnessing violence and neighborhood disadvantage manifests early in developmental pathways of risk, even before many youth initiate alcohol or cannabis use.

## Supplementary Material

Supplemental Tables

**Supplementary Information** The online version contains supplementary material available at https://doi.org/10.1007/s00127-025-02939-8.

## Figures and Tables

**Table 1:** Sample demographics by ADI quartile^a^

	Total sample *N* = 7332	Quartile 1 (*n* = 2758)	Quartile 2 (*n* = 2749)	Quartile 3 (*n* = 1181)	Quartile 4 (*n* = 944)

Race/ethnicity (%)					
Black	11.80	3.26	5.19	15.40	38.42
Latinx	25.13	20.22	26.35	28.10	27.77
White	63.07	76.52	68.46	56.50	33.81
Gender (%)					
Girl	45.53	42.31	46.14	45.19	46.55
Boy	52.33	51.41	51.72	52.79	50.84
Other	2.14	1.95	2.14	2.02	2.61
Age: M (SE)	12.94 (0.01)	12.96 (0.01)	12.93 (0.01)	12.95 (0.02)	12.90 (0.02)
Parental education (%)					
.≤ High school	4.43	2.11	4.16	3.92	9.99
High school	12.06	5.65	9.90	16.58	23.35
Some college	32.62	19.62	32.01	42.85	45.42
Bachelors degree	27.87	35.89	30.34	22.84	13.51
Post-graduate	23.02	36.73	23.59	13.81	7.73
Household income (%)					
≤ $50,000	36.72	17.28	29.93	49.69	72.39
$50,000-$99,999	32.59	27.32	38.57	36.06	24.26
≥ $100,000	30.69	55.40	31.50	14.26	3.35

a Accounting for nesting within families and sites and applying sample weights; M = mean; SE = standard error

ADI = Area Deprivation Index

**Table 2:** Past year (Follow-up 3) prevalence of witnessing violence and mean AEQ-AB and MEEQ-B scores, and lifetime cannabis and alcohol use by ADI quartile^a^

	Total sample *N* = 7,332	Quartile 1 (*n* = 2,758)	Quartile 2 (*n* = 2,749)	Quartile 3 (*n* = 1,181)	Quartile 4 (*n* = 944)	Significant^b^ differences in means

Witnessed violence (%; 95% CIs)	35.56 (24.34, 36.77)	34.66 (32.72, 36.61)	33.45 (31.44, 35.45)	37.48 (34.45, 40.51)	39.69 (36.27, 43.11)	Q1<Q4;Q2<Q4
Alcohol expectancies (M [95% CIs])						
Positive	0.05 (0.01, 0.03)	0.13 (0.09, 0.16)	0.05 (0.01, 0.08)	0.03 (−0.03, 0.09)	−0.03 (−0.09, 0.03)	Q1 > Q2; Q1 > Q3; Q1 > Q4; Q2 > Q4
Negative	0.10 (0.08, 0.12)	0.21 (0.18, 0.24)	0.14 (0.11, 0.18)	0.05 (0.00, 0.11)	−0.16 (−0.22, −0.09)	Q1 > Q2; Q1 > Q3; Q1 > Q4; Q2 > Q3; Q2 > Q4; Q3 > Q4
Ever sip alcohol	32.40 (31.19, 33.61)	38.22 (36.13, 40.30)	31.76 (29.77, 33.74)	28.97 (26.03, 31.90)	27.37 (24.15, 30.59)	Q1 > Q2; Q1 > Q3; Q1 > Q4; Q2 > Q4
Cannabis expectancies (M [95% CIs])						
Positive	0.06 (0.04, 0.09)	0.07 (0.03, 0.11)	0.05 (0.01, 0.09)	0.07 (0.01, 0.12)	0.08 (0.02, 0.15)	None
Negative	0.06 (0.04, 0.09)	0.19 (0.15, 0.22)	0.13 (0.10, 0.17)	−0.02 (−0.08, 0.03)	−0.20 (−0.27, −0.14)	Q1 > Q2; Q1 > Q3; Q1 > Q4; Q2 > Q3; Q2 > Q4; Q3 < Q4
Ever try cannabis	1.31 (0.10, 1.62)	0.71 (0.34, 1.08)	1.23 (0.74, 1.71)	1.76 (0.92, 2.61)	2.04 (1.00, 3.08)	Q1 < Q2; Q1 < Q3; Q1 < Q4

AEQ-AB = Alcohol Expectancy Questionnaire-Adolescent, Brief. MEEQ-B = Marijuana Effect Expectancies Questionnaire-Brief ADI = Area Deprivation Index. CI = confidence interval. Q = quartile.

a Accounting for nesting within families and sites and applying sample weights.

b Significant at *p* < 0.05, indicated by estimate falling outside the 95% CIs of the comparison group

**Table 3:** Results of regression analyses using witnessing violence as a predictor of AEQ-AB and MEEQ-B scores by ADI quartile^a^

	Standardized regression coefficient (95% CIs)				
	Quartile 1 (*n*=2758)	Quartile 2 (*n* = 2749)	Quartile 3 (*n* = 1181)	Quartile 4 (*n* = 944)	Significant ^b^ differences in regression coefficients

Alcohol expectancies					
Positive	0.12** (0.05, 0.19)	0.17*** (0.10, 0.24)	0.26*** (0.14, 0.37)	0.22** (0.10, 0.34)	Q1 < Q3
Negative	0.06 (0.00, 0.12)	0.01 (−0.06, 0.08)	0.10 (−0.01, 0.20)	0.08 (−0.05, 0.21)	None
Cannabis expectancies					
Positive	0.20*** (0.13, 0.28)	0.30*** (0.23, 0.38)	0.38*** (0.27, 0.49)	0.21** (0.09, 0.34)	Q1 < Q2; Q1 < Q3; Q3 > Q4
Negative	0.00 (−0.07, 0.06)	−0.08* (−0.16, -0.01)	−0.09 (−0.20, 0.01)	0.04 (−0.09, 0.16)	Q4 > Q3

AEQ-AB = Alcohol Expectancy Questionnaire-Adolescent, Brief. MEEQ-B = Marijuana Effect Expectancies Questionnaire-Brief (MEEQ-B). ADI = Area Deprivation Index. CI = confidence interval. Q = quartile. Models included race/ethnicity, gender, age, parental education, household income, and ever try cannabis (cannabis expectancies) or ever sip alcohol (alcohol expectancies).

a Accounting for nesting within families and sites and applying sample weights.

b Significant at *p* < 0.05, indicated by estimate falling outside the 95% CIs of the comparison group.

* *p* < 0.05;

** *p* ≤ 0.001;

*** *p* < 0.0001

## Data Availability

The data used for the current study are publicly available at https://abcdstudy.org/.
