# Supplementary material for: The association of witnessing violence with alcohol and cannabis expectancies among Black, Latinx, and White youth: considering neighborhood context
Source: Soc Psychiatry Psychiatr Epidemiol. 2025 Jul 2;60(11):2603–12. doi: 10.1007/s00127-025-02939-8 (PMC12551652; doi:10.1007/s00127-025-02939-8)
Supplement: Supplementary file 1 — Supplementary Material 1 [file 127_2025_2939_MOESM1_ESM.docx]

| Supplemental Table 1. Prevalence by ADI quartile of events defining witnessing violence ^a^ | | | | | |  |
| --- | --- | --- | --- | --- | --- | --- |
|  | Weighted frequencies (95% CIs) | | | |  |  |
|  | Quartile 1  (n=2,758) | Quartile 2  (n=2,749) | Quartile 3  (n=1,181) | Quartile 4  (n=944) | Significant ^b^ differences in weighted frequencies |  |
| Saw crime or accident | | 24.56  (22.81, 26.30) | 22.58  (20.78, 24.38) | 24.42  (21.76, 27.07) | 25.16  (22.05, 28.27) | Q1<Q2 |
| Saw or heard someone getting hit | | 16.74  (15.17, 18.31) | 17.29  (15.69, 18.89) | 21.40  (18.78, 24.03) | 23.31  (20.35, 26.28) | Q1<Q3; Q1<Q4; Q2<Q3; Q2<Q4; |
| Saw or heard someone being shot at (but not actually wounded) in your school or neighborhood | | 1.52  (1.06, 1.97) | 2.91  (2.26, 3.56) | 3.91  (2.69, 5.13) | 7.37  (5.70, 9.03) | Q1<Q2; Q1<Q3; Q1<Q4; Q2<Q4; Q3<Q4 |

ADI=Area Deprivation Index. CI=confidence interval. Q=quartile. ^a^ Accounting for nesting within families and sites and applying sample weights. ^b^ Significant at *p*<0.05, indicated by estimate falling outside the 95% CIs of the comparison group.

| Supplemental Table 2. Results of regression analyses using witnessing violence as a predictor of AEQ-AB positive expectancies score by ADI quartile ^a^ | | | | |  |
| --- | --- | --- | --- | --- | --- |
|  | Standardized regression coefficient (95% CIs) | | | |  |
|  | Quartile 1  (n=2,758) | Quartile 2  (n=2,749) | Quartile 3  (n=1,181) | Quartile 4  (n=944) |  |
| Witnessing violence | | 0.12*** | 0.17*** | 0.26*** | 0.33** |
| Race/ethnicity  (reference: White) | |  |  |  |  |
| Black | | -0.02 | -0.04 | -0.08 | -0.07 |
| Latinx | | -0.04 | -0.04 | -0.11 | 0.03 |
| Parental education  (reference: some college) | |  |  |  |  |
| < High school | | 0.14 | -0.03 | 0.04 | 0.01 |
| High school | | 0.06 | 0.05 | -0.07 | 0.00 |
| Bachelors degree | | 0.01 | 0.05 | -0.07 | 0.18* |
| Postgraduate | | 0.05 | 0.08 | -0.01 | 0.09 |
| Household income  (reference: $50,000-99,999) | |  |  |  |  |
| < $50,000 | | -0.12 | 0.00 | -0.05 | -0.04 |
| ≥ $100,000 | | -0.02 | 0.08 | 0.09 | -0.04 |
| Gender  (reference: boy) | |  |  |  |  |
| Girl | | 0.05 | 0.01 | 0.09 | 0.01 |
| Other gender | | 0.12 | 0.35 | 0.21 | 0.81*** |
| Age at time of interview | | 0.01*** | 0.01*** | 0.02*** | 0.01* |
| Ever sip alcohol | | 0.31*** | 0.34*** | 0.25*** | 0.32*** |

AEQ-AB=Alcohol Expectancy Questionnaire-Adolescent, Brief. ADI=Area Deprivation Index. CI=confidence interval. Q=quartile. ^a^ Accounting for nesting within families and sites and applying sample weights. * *p*<0.05; *** p*≤ 0.001;

*** *p*< 0.0001.

| Supplemental Table 3. Results of regression analyses using witnessing violence as a predictor of AEQ-AB negative expectancies score by ADI quartile ^a^ | | | | |  |
| --- | --- | --- | --- | --- | --- |
|  | Standardized regression coefficient (95% CIs) | | | |  |
|  | Quartile 1  (n=2,758) | Quartile 2  (n=2,749) | Quartile 3  (n=1,181) | Quartile 4  (n=944) |  |
| Witnessing violence | | 0.06 | 0.01 | 0.10 | 0.08 |
| Race/ethnicity  (reference: White) | |  |  |  |  |
| Black | | -0.39*** | -0.19* | -0.39*** | -0.26* |
| Latinx | | -0.26*** | -0.20*** | -0.26** | -0.25* |
| Parental education  (reference: some college) | |  |  |  |  |
| < High school | | -0.30* | -0.36** | 0.03 | -0.24 |
| High school | | -0.08 | -0.14 | -0.07 | -0.07 |
| Bachelors degree | | 0.05 | 0.05 | -0.02 | 0.14 |
| Postgraduate | | 0.07 | 0.04 | 0.15 | 0.13 |
| Household income  (reference: $50,000-99,999) | |  |  |  |  |
| < $50,000 | | 0.07 | -0.02 | 0.05 | -0.08 |
| ≥ $100,000 | | 0.04 | -0.02 | 0.04 | -0.04 |
| Gender  (reference: boy) | |  |  |  |  |
| Girl | | -0.01 | 0.00 | 0.01 | 0.00 |
| Other gender | | -0.11 | 0.01 | 0.11 | 0.02 |
| Age at time of interview | | 0.00* | 0.00 | 0.00 | 0.01 |
| Ever sip alcohol | | -0.08* | -0.06 | 0.02 | 0.03 |

AEQ-AB=Alcohol Expectancy Questionnaire-Adolescent, Brief. ADI=Area Deprivation Index. CI=confidence interval. Q=quartile. ^a^ Accounting for nesting within families and sites and applying sample weights. * *p*<0.05; *** p*≤ 0.001;

*** *p*< 0.0001.

| Supplemental Table 4. Results of regression analyses using witnessing violence as a predictor of MEEQ-B positive expectancies score by ADI quartile ^a^ | | | | |  |
| --- | --- | --- | --- | --- | --- |
|  | Standardized regression coefficient (95% CIs) | | | |  |
|  | Quartile 1  (n=2,758) | Quartile 2  (n=2,749) | Quartile 3  (n=1,181) | Quartile 4  (n=944) |  |
| Witnessing violence | | 0.20*** | 0.30*** | 0.37*** | 0.21** |
| Race/ethnicity  (reference: White) | |  |  |  |  |
| Black | | -0.16 | -0.10 | -0.27** | -0.20* |
| Latinx | | -0.04 | -0.07 | -0.25** | -0.09 |
| Parental education  (reference: some college) | |  |  |  |  |
| < High school | | -0.15 | -0.16 | -0.06 | -0.13 |
| High school | | 0.03 | 0.02 | -0.10 | -0.08 |
| Bachelors degree | | 0.04 | 0.05 | -0.08 | -0.01 |
| Postgraduate | | 0.08 | 0.09 | -0.03 | -0.05 |
| Household income  (reference: $50,000-99,999) | |  |  |  |  |
| < $50,000 | | -0.11 | 0.07 | 0.04 | 0.05 |
| ≥ $100,000 | | -0.05 | 0.04 | 0.02 | 0.00 |
| Gender  (reference: boy) | |  |  |  |  |
| Girl | | -0.02 | -0.08* | -0.01 | -0.06 |
| Other gender | | -0.01 | 0.29* | 0.17 | 0.61** |
| Age at time of interview | | 0.02*** | 0.00*** | 0.18*** | 0.02*** |
| Ever try cannabis | | 1.10*** | 1.00*** | 1.00*** | 0.64* |

MEEQ-B=Marijuana Effect Expectancies Questionnaire-Brief. ADI=Area Deprivation Index. CI=confidence interval. Q=quartile. ^a^ Accounting for nesting within families and sites and applying sample weights. * *p*<0.05; *** p*≤ 0.001;

*** *p*< 0.0001.

| Supplemental Table 5. Results of regression analyses using witnessing violence as a predictor of MEEQ-B negative expectancies score by ADI quartile ^a^ | | | | |  |
| --- | --- | --- | --- | --- | --- |
|  | Standardized regression coefficient (95% CIs) | | | |  |
|  | Quartile 1  (n=2,758) | Quartile 2  (n=2,749) | Quartile 3  (n=1,181) | Quartile 4  (n=944) |  |
| Witnessing violence | | 0.00 | -0.08* | -0.09 | 0.04 |
| Race/ethnicity  (reference: White) | |  |  |  |  |
| Black | | -0.28* | -0.13 | -0.18* | -0.10 |
| Latinx | | -0.13* | -0.08 | -0.72 | -0.14 |
| Parental education  (reference: some college) | |  |  |  |  |
| < High school | | -0.22 | -0.32* | -0.29 | -0.13 |
| High school | | 0.05 | -0.20* | -0.12 | -0.09 |
| Bachelors degree | | 0.08 | 0.08 | 0.07 | 0.15 |
| Postgraduate | | 0.07 | -0.01 | 0.11 | 0.21 |
| Household income  (reference: $50,000-99,999) | |  |  |  |  |
| < $50,000 | | -0.51 | -0.06 | 0.04 | -0.05 |
| ≥ $100,000 | | 0.07 | 0.02 | 0.12 | 0.18 |
| Gender  (reference: boy) | |  |  |  |  |
| Girl | | -0.09* | -0.04 | 0.00 | -0.02 |
| Other gender | | -0.16 | -0.21 | 0.01 | -0.38* |
| Age at time of interview | | 0.00 | 0.00 | -0.06 | -0.01* |
| Ever try cannabis | | -0.79** | -0.73*** | -0.45** | -0.30 |

MEEQ-B=Marijuana Effect Expectancies Questionnaire-Brief. ADI=Area Deprivation Index. CI=confidence interval. Q=quartile. ^a^ Accounting for nesting within families and sites and applying sample weights. * *p*<0.05; *** p*≤ 0.001;

*** *p*< 0.0001.
